# Closely related fungi employ diverse enzymatic strategies to degrade plant biomass

**DOI:** 10.1186/s13068-015-0285-0

**Published:** 2015-08-01

**Authors:** Isabelle Benoit, Helena Culleton, Miaomiao Zhou, Marcos DiFalco, Guillermo Aguilar-Osorio, Evy Battaglia, Ourdia Bouzid, Carlo P J M Brouwer, Hala B O El-Bushari, Pedro M Coutinho, Birgit S Gruben, Kristiina S Hildén, Jos Houbraken, Luis Alexis Jiménez Barboza, Anthony Levasseur, Eline Majoor, Miia R Mäkelä, Hari-Mander Narang, Blanca Trejo-Aguilar, Joost van den Brink, Patricia A vanKuyk, Ad Wiebenga, Vincent McKie, Barry McCleary, Adrian Tsang, Bernard Henrissat, Ronald P de Vries

**Affiliations:** Fungal Physiology, CBS-KNAW Fungal Biodiversity Centre and Fungal Molecular Physiology, Utrecht University, Uppsalalaan 8, 3584 CT Utrecht, The Netherlands; Microbiology and Kluyver Centre for Genomics of Industrial Fermentation, Utrecht University, Padualaan 8, 3584 CH Utrecht, The Netherlands; Megazyme International Ireland, IDA Business Park, Bray, Wicklow Ireland; Centre for Structural and Functional Genomics, Concordia University, 7141 Sherbrooke Street West, Montreal, QC H4B 1R6 Canada; Architecture et Fonction des Macromolécules Biologiques, Aix-Marseille Université, 13288 Marseille, France; CNRS, UMR7257, Aix-Marseille University, 13288 Marseille, France; Division of Microbiology and Biotechnology, Department of Food and Environmental Sciences, Viikki Biocenter 1, University of Helsinki, Helsinki, Finland; INRA, UMR1163 de Biotechnologie des Champignons Filamenteux, ESIL, Marseille, France; Department of Food Science and Biotechnology, Faculty of Chemistry, National University of México, UNAM, Cd. Universitaria, C.P. 04510 Mexico, DF Mexico; INRA, USC 1408 AFMB, 13288 Marseille, France; Department of Biological Sciences, King Abdulaziz University, Jeddah, Saudi Arabia

**Keywords:** *Aspergillus*, Enzyme production, Polysaccharides, Biofuel, Saccharification, Diversity, Plant biomass degradation

## Abstract

**Background:**

Plant biomass is the major substrate for the production of biofuels and biochemicals, as well as food, textiles and other products. It is also the major carbon source for many fungi and enzymes of these fungi are essential for the depolymerization of plant polysaccharides in industrial processes. This is a highly complex process that involves a large number of extracellular enzymes as well as non-hydrolytic proteins, whose production in fungi is controlled by a set of transcriptional regulators. *Aspergillus* species form one of the best studied fungal genera in this field, and several species are used for the production of commercial enzyme cocktails.

**Results:**

It is often assumed that related fungi use similar enzymatic approaches to degrade plant polysaccharides. In this study we have compared the genomic content and the enzymes produced by eight Aspergilli for the degradation of plant biomass. All tested Aspergilli have a similar genomic potential to degrade plant biomass, with the exception of *A. clavatus* that has a strongly reduced pectinolytic ability. Despite this similar genomic potential their approaches to degrade plant biomass differ markedly in the overall activities as well as the specific enzymes they employ. While many of the genes have orthologs in (nearly) all tested species, only very few of the corresponding enzymes are produced by all species during growth on wheat bran or sugar beet pulp. In addition, significant differences were observed between the enzyme sets produced on these feedstocks, largely correlating with their polysaccharide composition.

**Conclusions:**

These data demonstrate that *Aspergillus* species and possibly also other related fungi employ significantly different approaches to degrade plant biomass. This makes sense from an ecological perspective where mixed populations of fungi together degrade plant biomass. The results of this study indicate that combining the approaches from different species could result in improved enzyme mixtures for industrial applications, in particular saccharification of plant biomass for biofuel production. Such an approach may result in a much better improvement of saccharification efficiency than adding specific enzymes to the mixture of a single fungus, which is currently the most common approach used in biotechnology.

**Electronic supplementary material:**

The online version of this article (doi:10.1186/s13068-015-0285-0) contains supplementary material, which is available to authorized users.

## Background

Plant biomass is a highly attractive substrate for the production of biofuels and biochemicals as it is a renewable resource with a lower carbon footprint as compared to fossil substrates. It is also the predominant carbon source for most fungi and consists largely of polymeric compounds, of which polysaccharides are the main components [1, 2]. In addition, lignin encrusts the polysaccharides and acts as a physical barrier that impedes fungal enzymes from gaining access to them. Fungi cannot take up intact polysaccharides, but need to degrade them extracellularly to monomeric and oligomeric compounds using diverse enzymatic mixtures [1]. Plant polysaccharide degradation by fungi has been a topic of study for many decades due to its relevance in many industrial applications, such as paper and pulp, food and feed, beverages, textiles and detergents. The increasing interest in the production of alternative fuels and chemicals from plant biomass has provided an even greater push for research into fungal decomposition of plant biomass. In contrast to the earlier applications, production of biofuels and biochemicals would ideally involve complete depolymerization of the polysaccharides to monomers, putting a much higher demand on the efficiency of the enzymatic mixtures employed by industry.

Analysis of an increasing number of fungal genome sequences has demonstrated the fundamental differences in the plant polysaccharide degrading machinery of fungi [3–8]. In addition, the regulatory systems that control plant biomass degradation also differ strongly among fungi, although they are largely conserved among different *Aspergillus* species [9–16]. Results from a previous study on the utilization of polysaccharides by three Aspergilli [6] suggest that related fungal species may have developed different approaches to plant biomass degradation. In nature, biomass-degrading fungi live in mixed communities with other microorganisms. It can be expected that different species target distinct components of the substrate and degrade them using dissimilar enzyme combinations. An enhanced understanding of these strategies will not only increase our knowledge of fungal biodiversity, but will help in the design of efficient industrial enzyme mixtures for plant biomass degradation. In this study, we compared the plant biomass degradation potential and approaches of eight *Aspergillus* species: *A. clavatus*, *A. fischeri*, *A. flavus*, *A. fumigatus*, *A. nidulans*, *A. niger*, *A. oryzae* and *A. terreus* (Additional file 1: Table S1). The main aim was to evaluate if these related fungi have significant differences in their approach to degrade plant biomass and if this could provide leads to improve the saccharification efficiency of commercial enzyme cocktails. To do this we compared the genomic potential of these fungi as well as the enzymes sets they produce during growth on two common feedstocks, wheat bran and sugar beet pulp, that differ significantly in their composition (Table 1).

**Table 1:** Composition of the plant biomass substrates used in this study

	Rhamnose	Arabinose	Xylose	Mannose	Galactose	Glucose	Uronic acid	Polysaccharides
Wheat bran	0	17	35	1	2	42	3	Cellulose, (arabino) xylan
Sugar beet pulp	1	28	2	2	7	33	26	Cellulose, pectin, xyloglucan

Values are given in mol%. Polysaccharide composition is inferred from the monomer values.

## Results

### Genomic potential of the studied Aspergilli related to plant biomass utilization

**Fig. 1:**
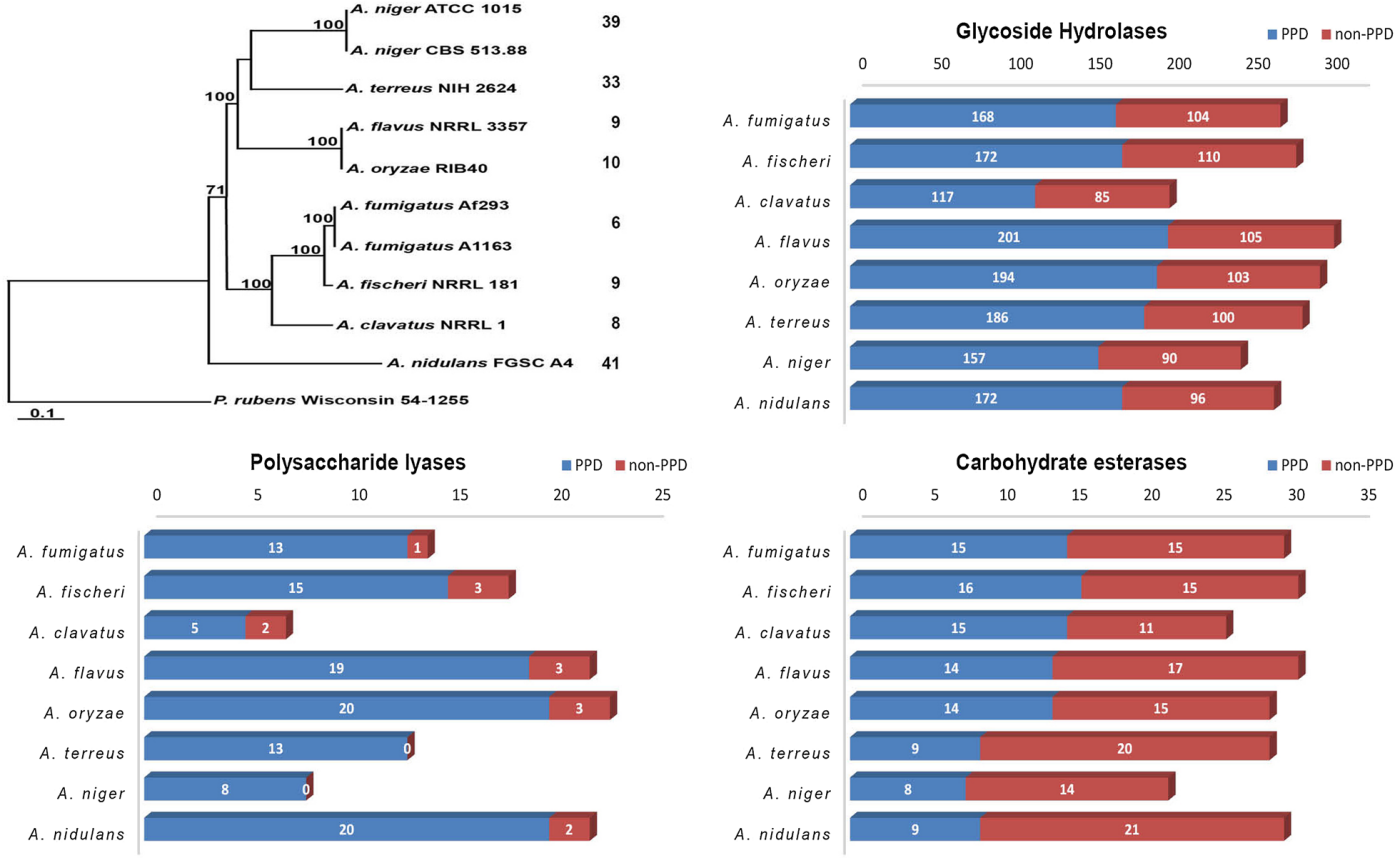
Taxonomic tree of the species used in this study and the numbers of glycoside hydrolases, polysaccharide lyases and carbohydrate esterases detected in their genomes. *PPD* plant polysaccharide degradation related. The *number* of unique genes per species is indicated behind their name in the taxonomic tree.

Based on the Carbohydrate-Active enZymes (CAZy) [17] annotation pipeline, total numbers of glycoside hydrolases (GH), polysaccharide lyases (PL) and carbohydrate esterases (CE) vary among the species (Fig. 1; Table 2). The percentage of GH genes related to plant polysaccharide degradation (PPD) is 58–66% for all genomes, except that *A. clavatus* has 20–30% less GH genes than the others (Fig. 1), largely due to a reduction in pectinases (GH28, GH54, GH78, GH88) (Table 2). *A. clavatus* also contains the lowest percentage of PPD-related PL genes (71% as compared to >86%), which are also all related to pectin degradation. The variations in CAZy content are relatively small compared to previous studies with a more diverse set of fungal species [3–8]. This can be explained by their close phylogenetic relationships and their similar habitats, which would push genome evolution in a similar direction.

**Table 2:** Comparison of the polysaccharide degradation potential of eight Aspergilli based on their genome content

Species	Cellulose^a^	Xyloglucan	Xylan	Galactomannan	Pectin	Starch	Inulin
	GH1, GH12^b^, GH5^b^, GH6, GH7, GH45, AA9	GH12^c^, GH29, GH31^d^, GH74, GH95	CE1^e^, CE15, GH3^f^, GH10, GH11, GH43^g^, GH62, GH67, GH115	GH2^h^, GH5^i^, GH26, GH27, GH36,	CE8, CE12, GH2^j^, GH28, GH35, GH43^k^, GH51, GH53, GH54, GH78, GH88, GH93, GH105, PL1, PL3, PL4, PL9, PL11	GH13^l^, GH15, GH31^m^	GH32
*A. nidulans*	22 (36)	7	29	19	71	21	2
*A. niger* ATCC1015	19 (33)	8	14	12	64	15	4
*A. terreus*	30 (43)	11	33	18	55	19	6
*A. oryzae*	22 (39)	7	34	14	89	23	4
*A. flavus*	22 (39)	7	34	14	92	22	4
*A. clavatus*	22 (28)	4	21	11	30	23	1
*A. fischeri*	30 (44)	8	29	14	66	24	2
*A. fumigatus* Af293	26 (37)	6	28	14	65	22	4

The potential per polysaccharide was determined by adding up the number of genes per polysaccharide-related (sub-) family.

^a^In brackets the numbers including putative GH3 BGLs are given. BGLs are also involved in other processes than cellulose degradation and their high number in the genomes could hide the real difference in gene numbers related to cellulose degradation between the species.

^b^Only endoglucanases of this family.

^c^Only xyloglucan-active endoglucanases of this family.

^d^Only α-xylosidases of this family.

^e^Only acetyl xylan esterases of this family.

^f^Only β-xylosidases of this family.

^g^Only β-xylosidases and α-arabinofuranosidases of this family.

^h^Only β-mannosidases of this family.

^i^Only endomannanases of this family.

^j^Only β-galactosidases of this family.

^k^Only endoarabinanases of this family.

^l^Only α-galactosidases and α-amylases of this family.

^m^Only α-galactosidases of this family.

When the *Aspergillus* genomes were compared for individual CAZy families, significant differences in numbers of genes were observed (Additional file 2: Table S2A, B). Variations in gene numbers are particularly obvious in certain CAZy families involved in the degradation of mannan (GH26), pectin (GH28, GH53, GH78, GH88, GH93, PL1, PL3, CE8 and CE12), xyloglucan (GH29 and GH74), starch (GH31), sucrose/inulin (GH32), cellulose (GH45 and AA9), and xylan (GH115 and CE15). Genes encoding lignin-modifying peroxidases are not present in any of these genomes, but significant differences are found in the number of laccases and other oxidoreductase enzymes, which may play a role in lignin or polysaccharide degradation (Additional file 2: Table S2B). *A. niger* is richest in laccases (13 in its genome), while the other species have two to nine.

Orthologous clustering of the CAZymes showed that only 14.7% of the genes encoding hydrolytic enzymes are shared by all species (Additional file 2: Table S3A). In contrast, 27.5% of the genes are unique to a single species, with the largest number in *A. nidulans*, *A. niger* and *A. terreus*. For the oxidative enzymes, 10.8% of the genes are shared by all species, while 40.8% of the genes are unique to a single species, with again the largest number in *A. nidulans*, *A. niger* and *A. terreus* (Additional file 2: Table S3B). In general, the CAZyme distribution among the species follows their phylogenetic relationship. In total, this means that only 70 genes are shared by all species, while the number of unique genes differs strongly by species (Fig. 1).

### Growth on plant biomass related substrates

**Fig. 2:**
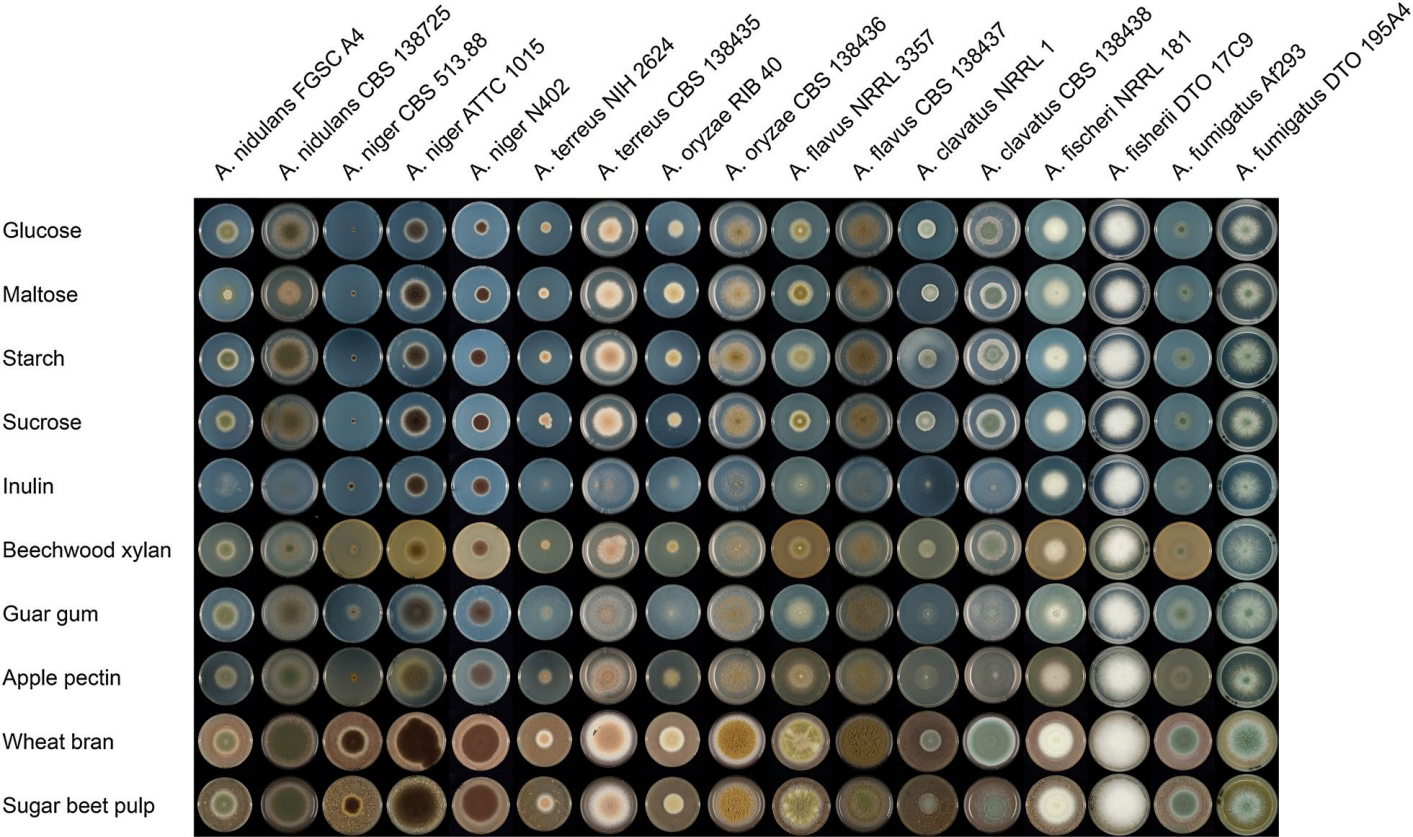
Growth profiling of eight Aspergilli on plant-biomass related carbon sources. A selection of the growth profile of the eight *Aspergillus* species on 35 plant biomass related carbon sources is presented. Minimal medium (MM) [1] was used supplemented with 25 mM of mono- or disaccharide, 1% polysaccharide or 3% crude plant biomass. Strains were grown for 5 days after which pictures were taken immediately.

Growth of the eight *Aspergillus* species was evaluated on 35 plant biomass related carbon sources (Fig. 2, full profiles are available at www.fung-growth.org). Two isolates per species were tested to check that the differences are species specific and not strain specific. The general growth speed differed between the strains of a species, but for most species no significant carbon source related differences were observed between the strains. An exception to this is *A. niger* CBS513.88 that grew poorly on all pure carbon sources and was shown to have an amino acid auxotrophy (unpublished data), which explains this phenotype. Apparently, both wheat bran (WB) and sugar beet pulp (SBP) contain sufficient protein/amino acids to supplement this deficiency. All other strains grew well on MM + glucose and glucose was therefore used as an internal reference to compare the strains, to avoid misleading differences caused by general differences in growth speed between the species. Growth on the other substrates relative to growth on glucose was then compared between the species.

Growth on pure cellulose was zero to very poor for all species. Most fungi had similar growth on glucose, maltose, starch and xylan, with the exception of *A. nidulans*, for which poor growth on maltose was observed for one strain. Growth on sucrose was similar to growth on glucose for nearly all strains, but strong differences were observed on inulin. Good growth on this substrate was observed for *A. niger* ATCC1015 and *A. fischeri*, while for all other species growth was reduced compared to sucrose. This does not correlate with the number of putative inulin-targeting genes in the genomes as *A. fischeri* has only two (Table 2), while *A. niger* has six just like *A. terreus*, which grows poorly on inulin. Good growth compared to the other species was observed for *A. niger*, *A. nidulans*, *A. fumigatus* and *A. flavus* on guar gum (galactomannan). While *A. nidulans* has the highest number of galactomannan-targeting genes (Table 2), *A. niger* has in fact the lowest number and *A. terreus* again grows poorly, even though it has the second highest number of galactomannan-targeting genes. Growth of *A. clavatus* was particularly poor on pectin which correlates well with its very low number of pectin-targeting genes (only 30 putative genes), which was less than half of the other species (Additional file 2: Table S2A, B).

All species grew well on wheat bran and also, with only *A. clavatus* having reduced growth, on sugar beet pulp. These substrates were therefore selected to analyze their enzymatic ability in more detail. Composition analysis (Table 1) showed that wheat bran contains mainly cellulose and (arabino)xylan, with xyloglucan and pectin as minor components. In contrast, sugar beet pulp contains mainly cellulose, xyloglucan and pectin, which explains the reduced growth of *A. clavatus*.

### Enzyme profiles during growth on wheat bran and sugar beet pulp

A preliminary test in which eight plant biomass degrading enzymes were measured at day two, three and four of cultivation, demonstrated that at day three activities were maximal for all fungi (data not shown). This time point was therefore selected for the full enzymatic analysis. Nineteen extracellular, lignocellulose-active enzyme activities of the liquid cultures were measured (Additional file 1: Figures S1–S3). Comparison of these profiles demonstrated strong differences among the species, not only in the quantities of the activities, but also in the induction of specific enzymes. For instance, the highest activity levels for most enzymes of *A. terreus* were observed during growth on sugar beet pulp, while wheat bran resulted in higher levels of most enzymes for *A. flavus* (Additional file 1: Figure S1). When the individual activities were compared across the species, specific differences became noticeable. Wheat bran consists mainly of cellulose and arabinoxylan and the main regulator controlling degradation of these polysaccharides is XlnR, which is present in all Aspergilli [18]. Endoxylanase and β-xylosidase were mainly produced on wheat bran, and levels were particularly high for *A. niger* (Additional file 1: Figure S1). Endoarabinanase, α-rhamnosidase, pectate lyase and endogalactanases, all related to pectin degradation, were mainly produced on sugar beet pulp, but rarely were all four activities produced by one species. Sugar beet pulp contains mainly cellulose and pectin and therefore pectinases and cellulases would be expected to be the main enzymes produced on this substrate, which is confirmed by our data.

**Fig. 3:**
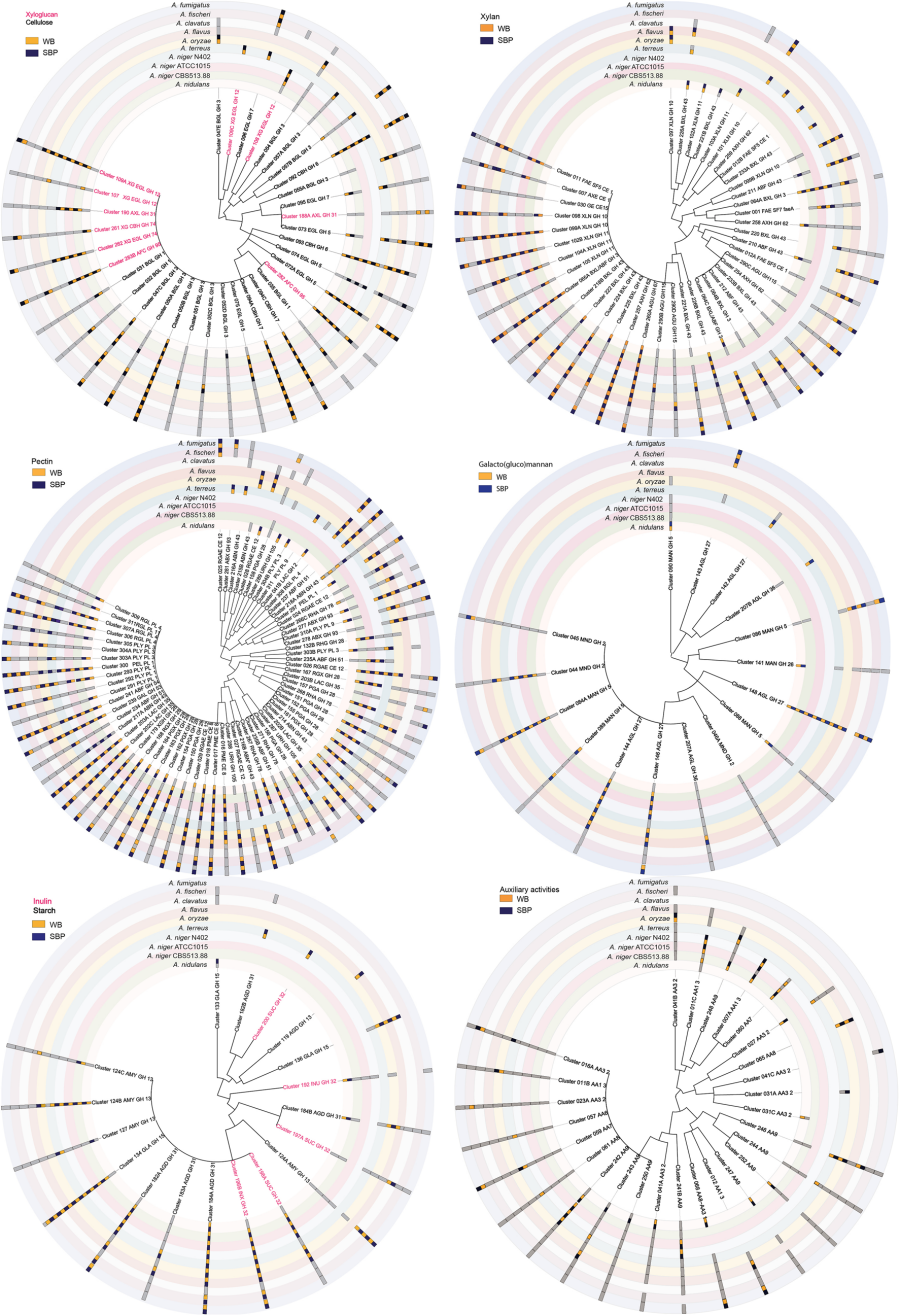
Proteins secreted by the eight *Aspergillus* species during growth on sugar beet pulp (SBP, *purple*) and wheat bran (WB, *orange*) as determined by mass spectrometry. Samples were taken after 3 days and are the same samples used for activity assays. The proteins are plotted using the ortholog clusters (Additional file 2: Table S3). Presence of the gene in a genome is depicted by a *grey box* in the *circle* corresponding to the species/strain.

Mass spectrometric analysis of the extracellular proteins confirmed the activity measurements with respect to the enzymes that were detected (Additional file 2: Table S4A–D). Figure 3 shows the presence of orthologous enzymes involved in the degradation of different polysaccharides in wheat bran and sugar beet pulp. This analysis demonstrates the high degree of diversity among the species in the production of orthologous enzymes. Only a few orthologous enzymes are produced by all or most species and in most cases they are produced on both wheat bran and sugar beet pulp (Fig. 3) although often with significantly different levels (Additional file 2: Table S4A–D). These data highlight the different enzymatic approaches used by the eight species to degrade plant biomass.

### Correlation of CAZy profiles, taxonomy and enzyme activity of the eight Aspergilli

**Fig. 4:**
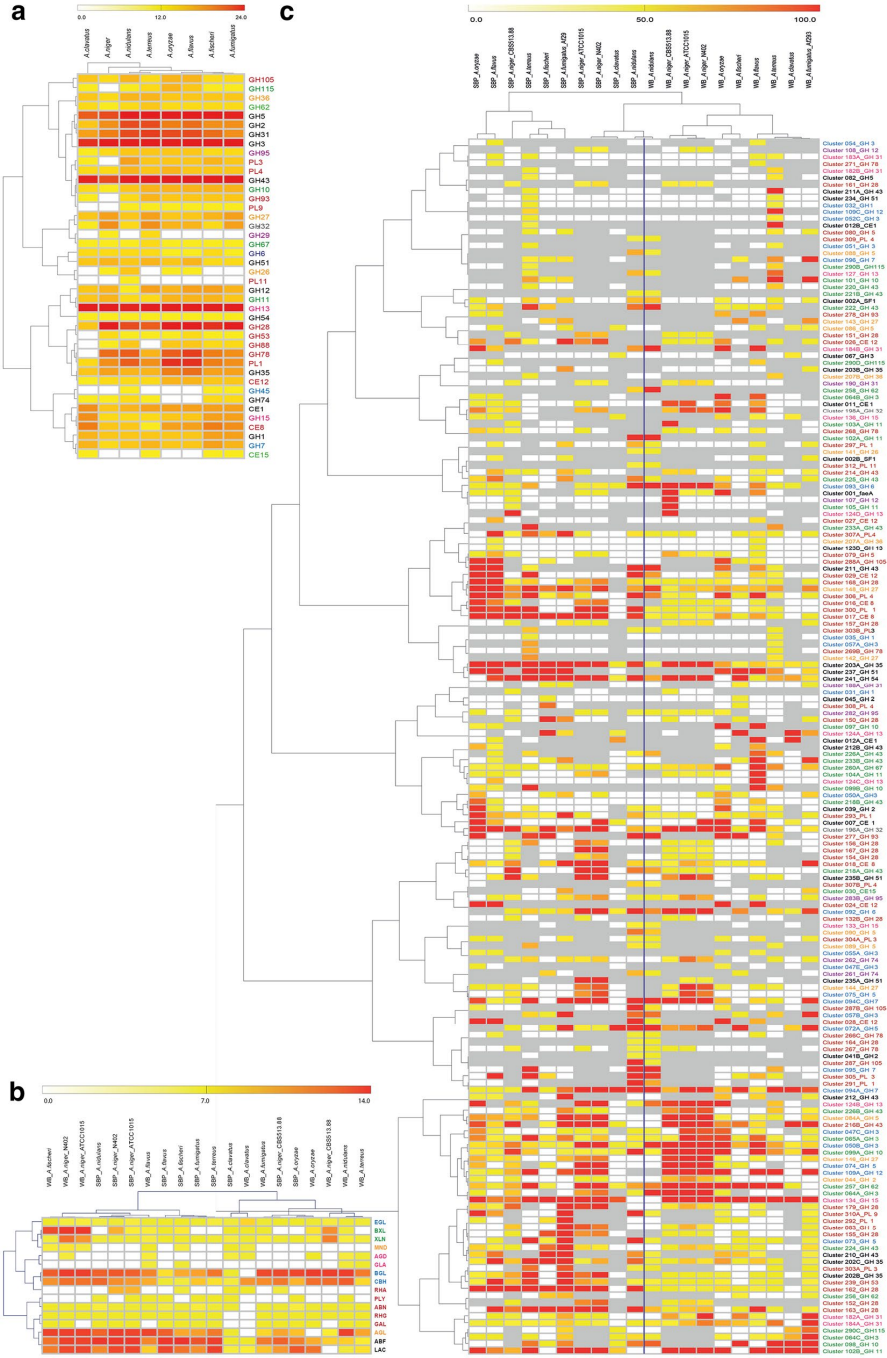
Correlation analysis of the genome (**a**), enzyme activity (**b**) and proteomics data (**c**).

Figure 4 shows the correlation of the species for genome content, enzyme activity and production of individual enzymes. Correlating the number of genes per CAZy family demonstrated that with respect to genome content, closely related species (*A. oryzae* – *A. flavus*, *A. fischeri* – *A. fumigatus*) cluster together (Fig. 4a). This indicates that the evolution of their genome content related to plant biomass degradation follows the evolutionary history of the species. However, more distantly related species, *A. nidulans* and *A. terreus*, can display similar CAZy content. A possible explanation for this finding is that natural habitat exerts a stronger influence on genome evolution than phylogenetic relatedness.

No clear correlation was observed between the enzyme activities produced in response to complex substrates and evolutionary relatedness (Fig. 4b), possibly due to the range of non-plant substrates some species are known to consume (e.g. collagen for *A. terreus*, *A. flavus* and *A. nidulans* and insect larvae for *A. clavatus*), resulting in a varying biotope range and dependence on plant biomass. The composition of wheat bran and sugar beet pulp is different and they should elicit different activity profiles. For six of the tested species, the wheat bran and sugar beet pulp activity profiles diverge strongly. Unexpectedly, the sugar beet pulp and wheat bran activity profiles clustered together for *A. flavus* and *A. oryzae*. Two of the three tested *A. niger* strains (N402 and ATCC 1015) clustered together for both substrates, while the third (CBS 513.88) was strongly divergent in the enzyme activity profile. These results show that strains of the same species (CBS 513.88 and ATCC 1015) with near identical genomic content can use significantly different sets of enzymes to hydrolyze complex biomass. It should be noted that genome sequence analysis suggests that strains ATCC 1015 and N402 are likely descended from the same isolate (A. Tsang and co-workers, unpublished data), which explains the clustering of their activity profiles.

Correlation of the proteomics data did not follow the activity correlation (Fig. 4b, c), which can be explained by the production of non-orthologous enzymes for the same general activity by different species (Additional file 2: Table S4A–D). This adds an additional dimension to the highly divergent strategies of these Aspergilli. Considering the fairly similar genome content of these species, we conclude that the differences in their plant biomass degrading strategies are mainly at the regulatory level. More detailed studies into the regulation of orthologous CAZyme encoding genes in several species could reveal whether this is due to different sets of target genes of the main regulators or whether additional unknown regulators modulate the influence of the main regulators.

## Discussion

In this study, we compared eight Aspergilli with respect to plant polysaccharide degradation. The variations in CAZyme content between these species were relatively low as compared to previous studies in which a more diverse set of fungal species was compared [3–8]. This can be explained by the close phylogenetic relationships and/or by and the highly similar habitats of these Aspergilli, which would direct genome evolution in a similar direction. Human use of and/or interaction with the species differs markedly; with *A. niger* and *A. oryzae* being widely used industrial fungi, *A. fumigatus* one of the most significant opportunistic fungal human pathogens, and *A. flavus* a plant pathogen. However, all these species are common inhabitants of soil and stored agricultural products, and their spores are widespread in both indoor and outdoor environments. Although some of the sequenced strains are domesticated and not recent natural isolates, the comparison to a second strain that is a natural isolate showed that growth on 35 carbon sources is nearly identical for two strains of the same species. This demonstrates that the sequenced isolates have maintained their natural ability to use the tested carbon sources.

Hierarchical clustering of the plant polysaccharide degrading enzymes of these species demonstrated that in general the species with the most similar CAZome are also taxonomically close.

The number of unique genes (Additional file 2: Table S2A, B) per species also correlates well with the phylogenetic distance of the species. The lowest number was found for *A. oryzae*, *A. flavus*, *A. fischeri* and *A. fumigatus*. As the first two and the last two species, respectively, are closely related, their high similarity explains this low number. The more distant species (*A. nidulans*, *A. terreus*, *A. niger*, *A. clavatus*) have higher numbers of unique genes.

A high level of variation was detected in the enzyme activities of the tested species during growth on sugar beet pulp and wheat bran. While all species grew well on these substrates, with the exception of somewhat less growth of *A. clavatus* on sugar beet pulp, the enzyme profiles of the species showed strong differences. Wheat bran consists mainly of cellulose and arabinoxylan, while sugar beet pulp contains mainly cellulose and pectin. Enzymes able to degrade these different combinations of polysaccharides would therefore be expected to be prominent in the culture filtrate of all species grown on these substrates. Our study confirmed this as nearly all enzyme activities detected on wheat bran in all species are involved in xylan and cellulose degradation, while mainly pectinolytic and cellulolytic enzymes were detected on sugar beet pulp. The main regulator controlling the production of xylanolytic and cellulolytic enzymes in *Aspergillus* is XlnR, which has been studied in detail in *A. niger*, *A. oryzae* and *A. nidulans* [19–28]. XlnR activates the expression of xylanolytic and cellulolytic genes in response to the presence of xylan or xylose, the latter being the actual inducer. Indications for similar regulation have been reported for the other species [29–35], although the range of target genes can differ per species [36, 37]. Regulation studies in *A. niger* have previously demonstrated that pectinolytic genes are induced by galacturonic acid, rhamnose, polygalacturonic acid or pectin [38–40]. Differences in pectinolytic gene content between *A. niger*, *A. nidulans* and *A. oryzae* may be influenced by the pH of their natural habitat [41]. An acidic pH favors pectin hydrolases, while a neutral to alkaline pH favors pectin lyases, supported by the finding that all fungal GH28 pectin hydrolases have activity optimum between pH 2 and pH 5, while pectin lyases have optimum between pH 7 and pH 10 (https://mycoclap.fungalgenomics.ca) [42]. The pH of most samples was 7 except for *A. nidulans* on sugar beet pulp (pH 8), *A. niger* on wheat bran (pH 5.5) and sugar beet pulp (pH 4.5), and *A. clavatus* on sugar beet pulp (pH 6). The pH in the sugar beet pulp cultures correlates well with the pectin hydrolase and lyase activities and with the proteomics results (Additional file 2: Table S4A–D). Therefore, the differences in enzyme levels are likely caused by regulatory variation. Since the major regulators are shared by all tested species [16], their function or range of target genes in the tested Aspergilli is different and/or additional non-shared regulators are involved in the utilization of complex biomass. A difference in the function of the arabinanolytic regulator AraR in *A. niger* and *A. nidulans* was recently described [43], and the inducers for activation of AmyR also appear to differ between *A. niger*, *A. nidulans* and *A. oryzae* [44–47]. More detailed analysis of the set of target genes, and function and mechanism of the polysaccharide related regulators in the other species will be required to understand the mechanism responsible for these differences. Interestingly, the production of several cellulases appears to be conserved among the species, suggesting that this may be a core-activity for all species. In contrast, the production of hemicellulases is highly varied, suggesting specific adaptations of the species in their biomass degrading approach.

Laccase activity was detected for most species, with the highest activity on wheat bran for *A. flavus* and on sugar beet pulp for *A. fumigatus*. This does not correlate with the numbers of putative laccases detected in the genomes, suggesting significantly different regulation of the production of these enzymes among the species. Induction of laccase-encoding genes has been mainly studied in basidiomycetes in which transcription is modulated by metal ions (Cu^2+^, Ag^+^, Mn^2+^), aromatic compounds, nitrogen and carbon sources (nature and ratio) [48]. In ascomycetes, regulatory elements such as heat shock elements, metal response elements and nitrogen metabolite regulation elements (NIT-2 like) were identified in the promoter region of laccase-like multicopper oxidase [49]. In addition, laccases are also involved in other biological processes, such as spore pigment formation [50], and not only in lignin degradation, so the total number of laccases likely does not reflect the number of laccases which play a role in plant biomass degradation.

Although the fungi tested in this study produce diverse enzyme sets, they all grow well on the crude plant biomass substrates. This suggests that different strategies for the degradation of plant biomass may be equally efficient (as measured by fungal growth). In biotechnological applications, such as biofuel production, complete hydrolysis of the plant biomass is difficult to achieve with currently available enzyme cocktails. This may in part be explained by the absence of specific activities in these mixtures. The data obtained in this study show the existence of distinctly different enzymatic approaches to degrade biomass. A judicious mix of these approaches is likely to result in improved enzyme cocktails for biomass hydrolysis. Recently it was shown that addition of *Podospora anserina* hydrolases increases the efficiency of a *Trichoderma reesei* enzyme mixture [51]. In this study we provide indications that similar results could be obtained with more closely related fungi. The advantage of using enzymes from other Aspergilli to improve enzyme cocktails of *A. niger* or *A. oryzae* is that heterologous production of these enzymes is not likely to cause problems due to the high similarity in gene structure of these species.

## Conclusions

In this study we demonstrated that closely related fungi use highly diverse enzymatic strategies for the degradation of the same substrates, but with similar efficiency. It can be assumed that in nature not all fungi target the same parts of the substrate. Therefore the identification of the enzyme sets employed by different fungi can be used to design efficient commercial enzyme cocktails by combining these enzyme sets. This could significantly improve the saccharification efficiency of industrial enzyme cocktails. Combining the complete enzymes set of two fungi is likely to improve saccharification efficiency more than adding specific enzymes to the cocktail produced by a single species.

## Methods

### Media, growth conditions and chemicals

The fungal strains used in this study are listed in Additional file 1: Table S1. *Aspergillus* minimal medium was described previously [52]. All monomeric and oligomeric carbon sources were added to a final concentration of 25 mM, while pure polymeric substrates and crude substrates were added to a final concentration of 1 and 3%, respectively. The pH of the medium was adjusted to 6.0. For plate growth, the center of the plates was inoculated with 2 μl of a suspension of 500 spores/μl and plates were incubated for 5 days at 30°C. All eight species were grown on minimal medium with 35 carbon sources including crude plant biomass, pure plant polysaccharides, oligosaccharides, monosaccharides and control substrates (casein, lignin) (Additional file 1: Figure S1, www.fung-growth.org). To confirm that the detected differences were species specific, a second isolate of each species was examined along with the sequenced strain. Growth on 25 mM glucose was used as a reference because the tested strains grow at different rates and glucose, among the monosaccharides, supported the fastest growth for all species. Growth on the other substrates relative to growth on glucose was then compared among the species. Growth on plates was analyzed by visual inspection by two authors independently after which these were compared and discussed.

Liquid cultures were inoculated with 10^6^ spores/ml (final concentration) and incubated at 250 rpm for 3 days. All cultures were incubated at 30°C and performed in duplicate. Two to three strains of all species were grown in liquid cultures with 1% wheat bran or 1% sugar beet pulp. Culture filtrates after 3 days of cultivation were analyzed for the presence of free monomeric sugars, but no glucose, xylose, galacturonic acid, rhamnose or fructose was detected. SDS-PAGE analysis of the extracellular proteins revealed nearly identical profiles for strains of the same species (Additional file 1: Figure S6), indicating that enzyme production is highly conserved within a species. Detailed analysis of the produced enzymes was therefore only performed on a single strain.

Glucose, maltose, sucrose, inulin, beechwood xylan, Guar gum, apple pectin and all *p*-nitrophenyl-substrates were from Sigma–Aldrich. Soluble starch was from Difco. Red Debranched Arabinan (S-RDAR), Azo-CM-cellulose (S-ACMC), Azo-galactan (S-AGALP) and AZ-rhamnogalacturonan (S-AZRH), Azo-wheat arabinoxylan (S-AWAXP) and polygalacturonic acid (PGA) were from Megazyme International Ireland.

### Composition analysis of plant biomass substrates

Sugar composition was determined by analyzing the sugars as their alditol acetate derivatives using GC-FLD as described previously [53].

### CAZy annotation

The identification step of CAZymes followed the procedures previously described [6] where sequences are subject to BlastP analysis [54] against a library composed of modules derived from the CAZy database, the positive hits are then subjected to a modular annotation procedure that maps the individual modules onto the peptide using hits against libraries of catalytic and carbohydrate models derived from CAZy using BlastP or Hidden Markov models [54, 55]. The functional annotation step involves BlastP comparisons against a library of modules derived from biochemically characterized enzymes [6].

### Orthology and synteny analysis

Genome scale protein ortholog clusters were constructed using OrthoMCL [56] by inflation factor 1, *E* value cutoff 1E−3, percentage match cutoff 60% as for identification of distant homologs [57]. The orthologs clusters were further split according to the synteny detected by the Sybil algorithm [58] at www.aspgd.org. Sequences of genes were manually double checked by multiple sequence alignments with MAFFT [59] and potential errors of gene models were corrected.

### Enzyme assays

All exo-acting CAZy enzyme activities were performed in microtiter plates. Reactions were carried out in 100 µl volumes containing 25 mM sodium acetate (pH 5), 0.01% substrate and suitably diluted culture filtrate. The mixture was incubated at 30°C for 2 h and the reaction was terminated by the addition of 100 µl 250 mM sodium carbonate. Enzyme activities (α-arabinofuranosidase, cellobiohydrolase, α-galactosidase, β-galactosidase, α-glucosidase, β-glucosidase, glucoamylase (α-maltosidase), β-mannosidase, α-rhamnosidase and β-xylosidase) were determined spectrophotometrically at 405 nm by measuring the release of *p*-nitrophenol (*p*NP) from their appropriate *p*NP-substrates and standardized against a known concentration of *p*-nitrophenol (*p*NP). Activities were expressed as nmol *p*NP/ml sample/min.

Endoarabinanase, endo-1,4-β-glucanase (cellulase), endo-1,4-β-galactanase and rhamnogalacturonanase activities were measured using 20 mg/ml of Red Debranched Arabinan (S-RDAR), Azo-CM-cellulose (S-ACMC), Azo-galactan (S-AGALP) and AZ-rhamnogalacturonan (S-AZRH), respectively. Endo-1,4-β-xylanase activity was measured using 10 mg/ml Azo-wheat arabinoxylan (S-AWAXP). 100 μl reactions were carried out containing equal volumes of buffered substrate (pH 4.5) and suitably diluted culture filtrate which were then incubated at 40°C for 1 h in the case of the endoarabinanase, endo-1,4-β-glucanase and endo-1,4-β-xylanase activities and 16 h for the endo-1,4-β-galactanase and rhamnogalacturonan activities. Endoarabinanase reactions were terminated with the addition of 400 μl 95% ethanol, endo-1,4-β-galactanase, rhamnogalacturonanase and endo-1,4-β-xylanase reactions with 250 μl 95% ethanol and endo-1,4-β-glucanase reactions with a 250 μl solution of sodium acetate trihydrate (40 mg/ml) and zinc acetate (4 mg/ml) in 76% ethanol. Precipitated reactions were then centrifuged at 1,000×*g* for 10 min and optical density of supernatants was measured at 590 nm. Endoarabinanase reactions were measured at 520 nm. Endo-acting enzyme activities are expressed as amount of dye released (absorbance change)/ml sample/min.

Pectate lyase activity was assayed using polygalacturonic acid (PGA). Reaction mixtures contained equal volumes of 50 mM *N*-cyclohexyl-3-aminopropanesulfonic acid (CAPS) (pH 10.0) and 2.5 mg/ml PGA, to which suitably diluted culture filtrate was added. Changes in absorbance at 235 nm were measured for approximately 30 min at 40°C.

Laccase activity was assayed using 2,2′-azino-di-(3-ethylbenzothiazoline-6-sulphonic acid) (ABTS). Reaction mixtures contained 700 µl H_2_O, 100 µl 0.5 M glycine–HCl (pH 3.0), 100 µl culture filtrate and 100 µl 14 mM ABTS. The reaction was monitored by measuring the change in absorption at 436 nm at 30°C. The extinction coefficient of 29,300/M/cm was used for oxidized ABTS. Activity is expressed as is in nmol/min/ml.

Feruloyl esterase activities were determined spectrophotometrically (Shimadzu PharmaSpec UV-1700) at 37°C in 100 mM MOPS (3-(*N*-morpholino)propanesulfonic acid) buffer (pH 6.0). Methyl caffeate (MC), methyl ferulate (MF), methyl *p*-coumarate (MpC) and methyl sinapate (MS) (1.18 mM stock solutions in 100 mM MOPS, pH 6.0) were used as substrates. Reaction mixture contained 100 µl of culture liquid, 870 µl MOPS (3-(*N*-morpholino)propanesulfonic acid) buffer and the reaction was started by the addition of 30 µl substrate. Absorbance was monitored for 5 min at 308 nm for MpC (ε_308_ = 20,390/M/cm), 320 nm for MF (ε_320_ = 29,680/M/cm) and MS (ε_320_ = 15,890/M/cm), and 322 nm for MC (ε_322_ = 14,720/M/cm). FAE activities were expressed as nkat/l (10^−9^ mol/s/l).

### Determination of monomeric sugars in the cultures

Presence of monomeric sugars in wheat bran and sugar beet pulp liquid cultures was measured by using Megazyme’s Assay kits for glucose and fructose (K-FRUGL), xylose (K-XYLOSE), glucuronic acid (K-URONIC) and rhamnose (K-RHAMNOSE) using the provider’s instruction. All measurements were done with two biological replicates.

### SDS-page

Protein profiles were obtained by combining 25 μl of culture supernatant supplemented with 5 μl of 5 × Laemmli Loading Buffer (50 mM Tris–HCl pH 6.8, 2% SDS, 10% glycerol, 0.1 M dithiothreitol, 0.2 mg/ml Bromophenol Blue) and separating this on 12% SDS-PAGE gels. Proteins were visualized by silver staining and a PageRuler Unstained Protein Ladder (Thermo Scientific) was used as protein marker.

### Proteomics analysis

Proteins from 3 ml of culture filtrate were precipitated with cold TCA/acetone and the amount of protein recovered was determined using the RCDC kit assay (BioRad, Mississauga, ON, Canada). Five micrograms of protein were digested with trypsin and an aliquot analyzed by LC–MS/MS as previously described [60] on a Velos LTQ-Orbitrap mass spectrometer (Thermo-Fisher, San Jose, CA, USA). MS/MS data were processed using Proteome Discoverer Quant 1.3 (Thermo-Fisher) and spectral data were searched against *Aspergillus* protein databases downloaded from the Aspergillus Genome Database (AspGD). Search parameters used were 0.80 Da for fragment ion tolerance and 10.0 ppm for parent ion tolerance, fixed iodoacetamide cysteine modification and variable methionine oxidation. Protein and peptide identification confidence filters were applied to satisfy a 1% false discovery rate at the Peptide and Protein level. Protein grouping was applied so as to satisfy the principles of parsimony. The mass spectrometry proteomics data have been deposited to the ProteomeXchange Consortium (http://proteomecentral.proteomexchange.org) via the PRIDE partner repository with the dataset identifier PXD000982.

### Hierarchical clustering and correlation analysis

Matrix files of presence/absence and activity of CAZyme encoding genes and protein production measured by proteomics experiments were generated. The hierarchical clustering of CAZyme encoding genes were created using R [61] using the Euclidean distance with complete linkage and visualized by iTOL [62, 63]. The dendrogram and heatmaps of protein abundance were created and visualized using Genesis [64] with Pearson’s correlation and complete linkage.

### Phylogenetic analysis

Sequences of the RPB1, RPB2 (RNA polymerase II genes), Tsr1 (putative ribosome biogenesis protein), Cct8 (putative chaperonin complex component TCP-1) and AguA (α-glucuronidase) genes were downloaded from the full genome data sets and aligned using the Muscle software in the MEGA5 package [65]. After aligning, the data sets were combined and maximum likelihood analysis was performed using RAxML version 7.2.8 [66]. Each locus was treated as a separate partition. The number of bootstrap replicates was set on 1,000 replicates. Sequences of *Penicillium chrysogenum* Wisconsin 54–1,255 were used as outgroup.

## Additional files

Additional file 1: Combines Additional File Figures S1–S4 and Table S1. Figure S1: Hydrolytic enzyme activity profiles of the eight species. Figure S2: Laccase activity of the eight species. Figure S3: Differences in feruloyl esterase production. Figure S4: Conserved SDS-PAGE profiles for isolates of the same species. Table S1. Strains used in this study. Additional file 2: Combines Additional file Tables S2–S4 (excel format). Table S2A. Numbers of putative genes per CAZy family for the 10 genomes addressed in this study. Table S2B. Numbers of putative genes per Plant Polysaccharide Degradation-related CAZy family for the 10 genomes addressed in this study. Table S3A. Orthology clusters of feruloyl esterase (SF), glycoside hydrolase (GH), carbohydrate esterase and polysaccharide lyase (PL) families. Table S3B. Orthology clusters of auxiliary activities (AA). Table S4A. Detection of proteins in cultures grown on wheat bran sorted by CAZy family. Table S4B. Detection of proteins in cultures grown on sugar beet pulp sorted by CAZy family. Table S4C. Detected proteins in cultures grown on wheat bran sorted by number of species that contain an orthologue. Table S4D. Detected proteins in cultures grown on sugar beet pulp sorted by number of species that contain an orthologue.

## Abbreviations

ABTS: 2,2′-azino-di-(3-ethylbenzothiazoline-6-sulphonic acid)
CAPS: *N*-cyclohexyl-3-aminopropanesulfonic acid
CE: carbohydrate esterase
GH: glycoside hydrolase
MC: methyl caffeate
MF: methyl ferulate
MM: minimal medium
MOPS: 3-(*N*-morpholino)propanesulfonic acid
MpC: methyl *p*-coumarate
MS: methyl sinapate
PGA: polygalacturonic acid
PL: polysaccharide lyase
*p*NP: *p*-nitrophenol
PPD: plant polysaccharide degradation
S-ACMC: Azo-CM-cellulose
S-AGALP: Azo-galactan
S-AZRH: AZ-rhamnogalacturonan
S-AWAXP: Azo-wheat arabinoxylan
S-RDAR: Red Debranched Arabinan
SBP: sugar beet pulp
WB: wheat bran
