# Supplementary material for: Closely related fungi employ diverse enzymatic strategies to degrade plant biomass
Source: Biotechnol Biofuels. 2015 Aug 1;8:107. doi: 10.1186/s13068-015-0285-0 (PMC4522099; doi:10.1186/s13068-015-0285-0)
Supplement: Additional file 1: — Combines Additional File Figures S1–S4 and Table S1. Figure S1: Hydrolytic enzyme activity profiles of the eight species. Figure S2: Laccase activity of the eight species. Figure S3: Differences in feruloyl esterase production. Figure S4: Conserved SDS-PAGE profiles for isolates of the same species. Table S1. Strains used in this study. [file 13068_2015_285_MOESM1_ESM.pdf]

Supplemental Figures 1-4 and Supplemental Table 1

Contents

Supplemental Figure 1: Hydrolytic enzyme activity profiles of the eight species 2
Supplemental Figure 2: Laccase activity of the eight species 4
Supplemental Figure 3: Differences in feruloyl esterase production 5
Supplemental Figure 4: Conserved SDS-PAGE profiles for isolates of the same species 6
Supplemental Table 1. Strains used in this study 7
References 8

Supplemental Figure 1: Hydrolytic enzyme activity profiles of the eight species

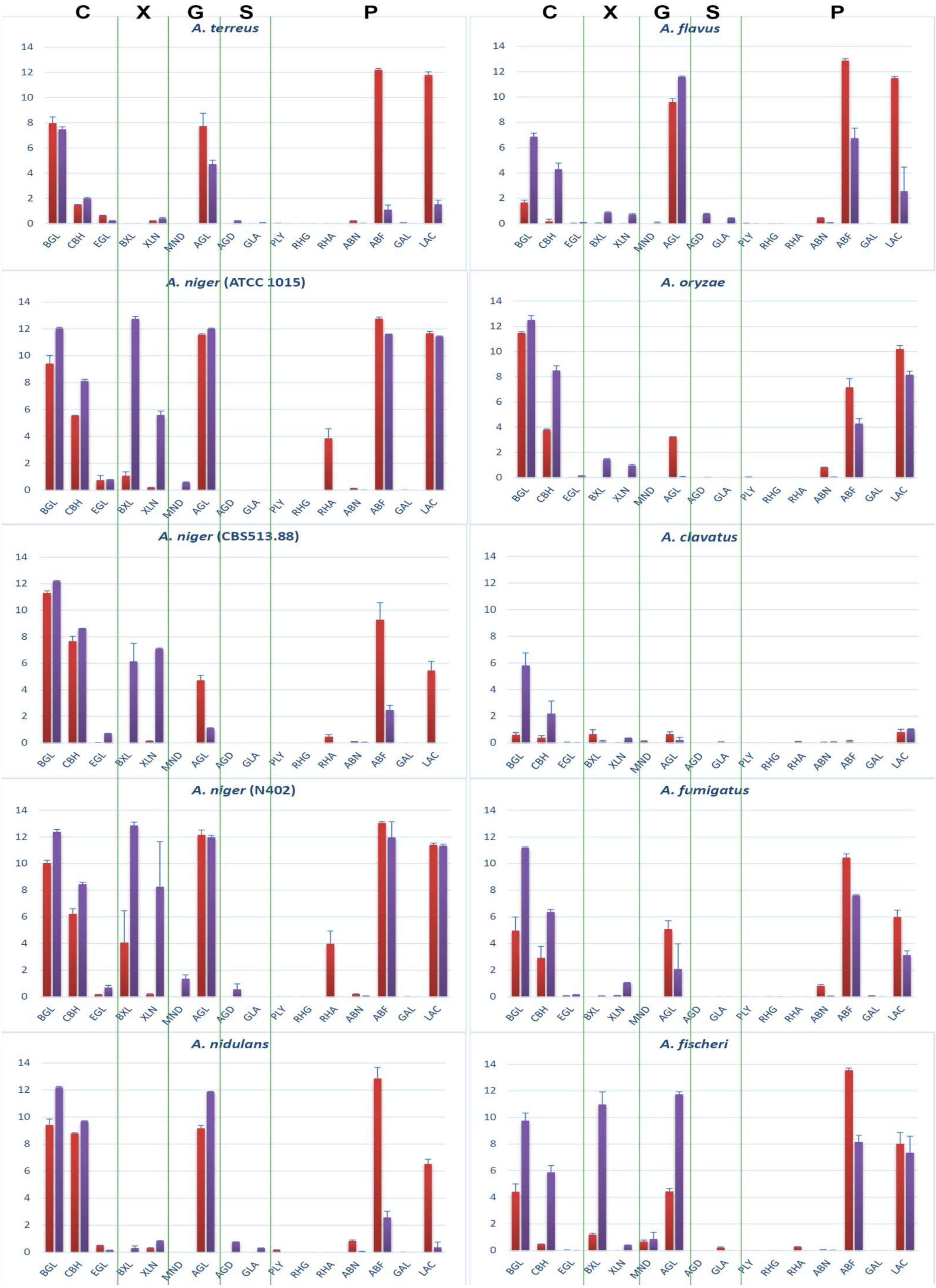

Enzyme profiles of the eight *Aspergillus* species during growth on sugar beet pulp (SBP, red) and wheat bran (WB, blue). Samples were taken after three days and are identical to the samples used for proteomics. Vertical lines separate the activities related to the same substrate; from left to right: cellulose (C), xylan (X), galactomannan (G), starch (S), pectin (P). BGL = β-glucosidase, CBH = cellobiohydrolase, EGL = endoglucanase, BXL = β-xylosidase, XLN = endoxylanase, MND = β-mannosidase, AGL = α-galactosidase, AGD = α-glucosidase, GLA = glucoamylase, PLY = pectate lyase, RHG = endorhamnogalacturonase, RHA = α-rhamnosidase, ABN = endoarabinanase, ABF = α-arabinofuranosidase, GAL = endogalactanases, LAC = β-galactosidase.

Activity units were: For all *exo*-acting enzyme activities (ABF, CBH, AGL, LAC, AGD, BGL, GLA, MND, RHA, BXL) are expressed as nmol *p*NP released/ml sample/min. *Endo*-acting enzyme activities (ABN, EGL, GAL, XLN and RHG) are expressed as amount of dye released (absorbance change)/ml sample/min. Pectate lyase (PLY) activity is expressed as absorbance change/ml sample/min.

Significant differences can be observed between the profiles both with respect to the relative activity of the different enzymes as well as to the production of the different activities on SBP and/or WB. Related species do not have high similarity with respect to enzyme activity profiles. Significant differences were observed between *A. oryzae* and *A. flavus*, and between *A. fumigatus* and *A. fischeri*. The reduction of pectinase encoding genes in the *A. clavatus* genome is reflected in very low pectinolytic enzyme activity.

Supplemental Figure 2: Laccase activity of the eight species

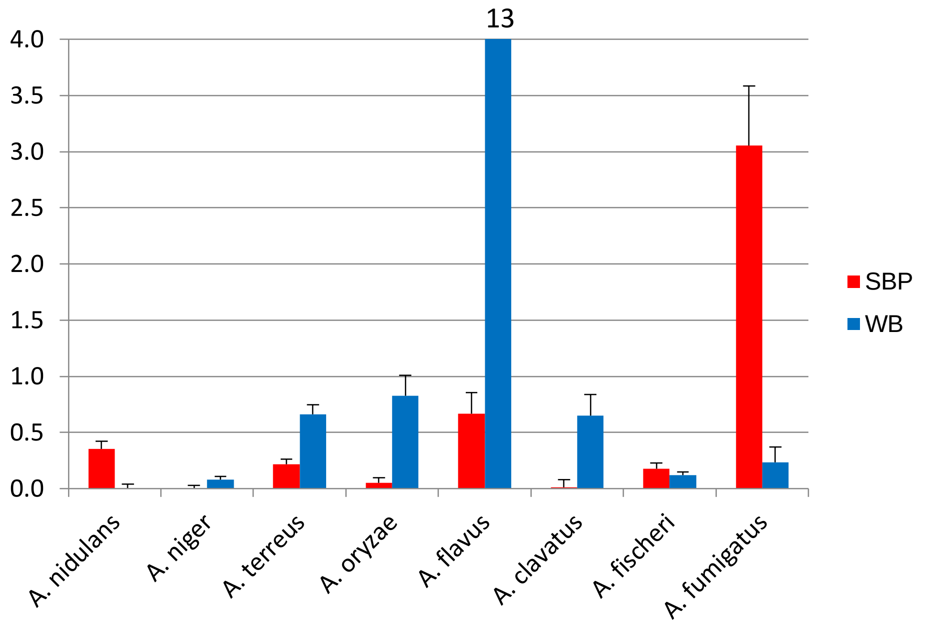

Laccase activity of the eight *Aspergillus* species during growth on sugar beet pulp (SBP, red) and wheat bran (WB, blue). Samples were taken after three days and are identical to the samples used for proteomics. Laccase activity is in nmol/min/ml. *A. niger* N402 was used for these assays.

The highest laccase activity was observed for *A. flavus* during growth on WB and for *A. fumigatus* during growth on SBP. Activities in the other species were significantly lower.

Supplemental Figure 3: Differences in feruloyl esterase production

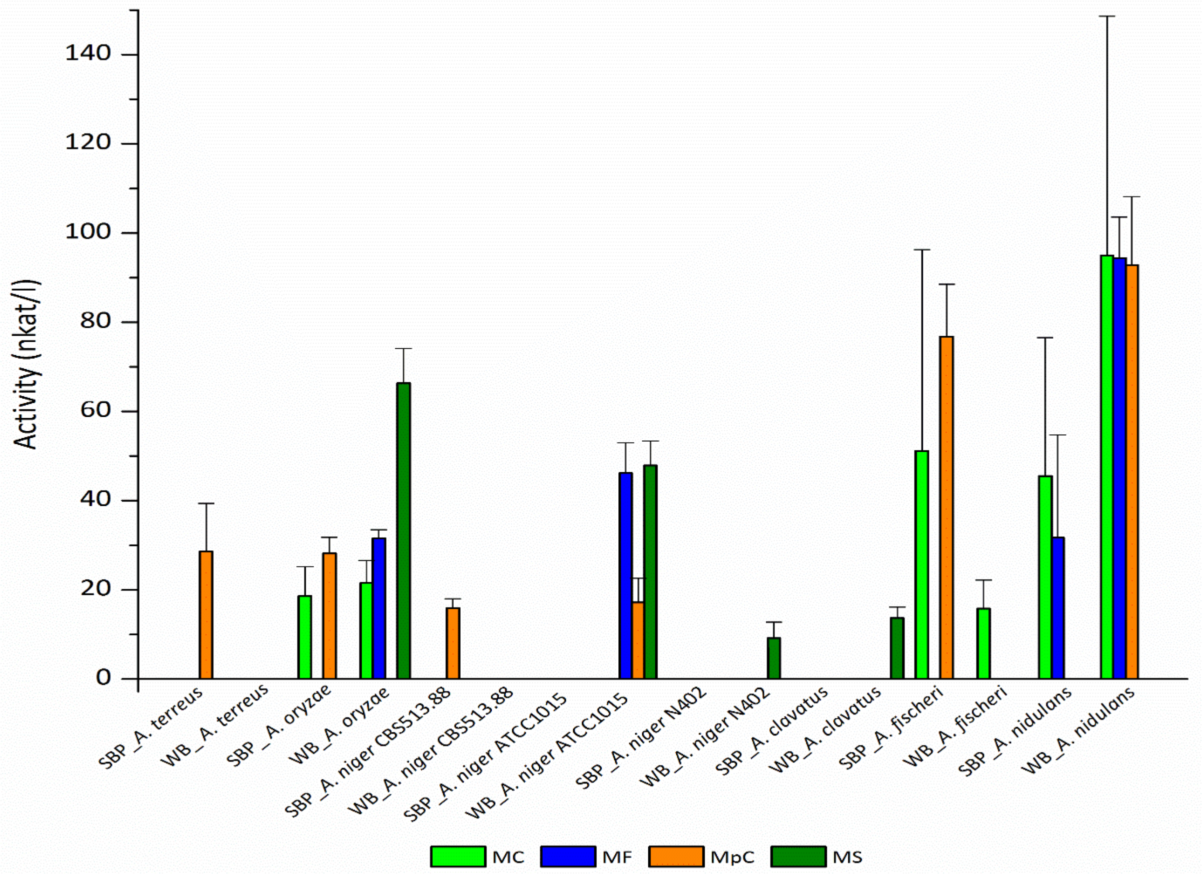

Fungal strains were grown in 50 ml liquid minimal medium [1] with 1% wheat bran (WB) or sugar beet pulp (SBP) in 250 ml Erlenmeyer flasks. Culture filtrate samples were taken on day 3 and used for enzyme assays. Feruloyl esterase activities were determined spectrophotometrically at 37 °C in 100 mM MOPS buffer (pH 6) using methyl caffeate (MC), methyl ferulate (MF), methyl *p*-coumarate (MpC) and methyl sinapate (MS) as substrates. Absorbance was monitored for 5 min at 308 nm for MpC (ε_{308} = 20,390 M^{-1} cm^{-1}), 320 nm for MF (ε_{320} = 29,680 M^{-1} cm^{-1}) and MS (ε_{320} = 15,890 M^{-1} cm^{-1}), and 322 nm for MC (ε_{322} = 14,720 M^{-1} cm^{-1}).

Strongly divergent FAE activity profiles were observed for the studied Aspergilli. Differences were observed with respect to the carbon source that induced the activities as well as the substrate that was converted in the assays. The highest FAE activities were detected in *A. nidulans* WB cultures, but no activity was observed against MS. Both *A. terreus* and *A. niger* CBS513.88 strains produced FAE activity only in SBP cultures with M*p*C as substrate. In contrast, *A. niger* N402 and *A. clavatus* produced FAE activity only in the WB cultures with MS as a substrate. While no activity was detected in the SBP cultures of *A. niger* ATCC1015, it produced activities against MF, M*p*C and MS in WB cultures. FAE activity against MC was detected in both SBP and WB cultures of *A. oryzae*, *A. fischeri* and *A. nidulans*. For other substrates, the activity profiles differed between these strains. As it is unlikely that the substrate specificity of orthologous enzymes would differ this much with respect to these four substrates, the data implies that different feruloyl esterases are produced by the strains.

Supplemental Figure 4: Conserved SDS-PAGE profiles for isolates of the same species

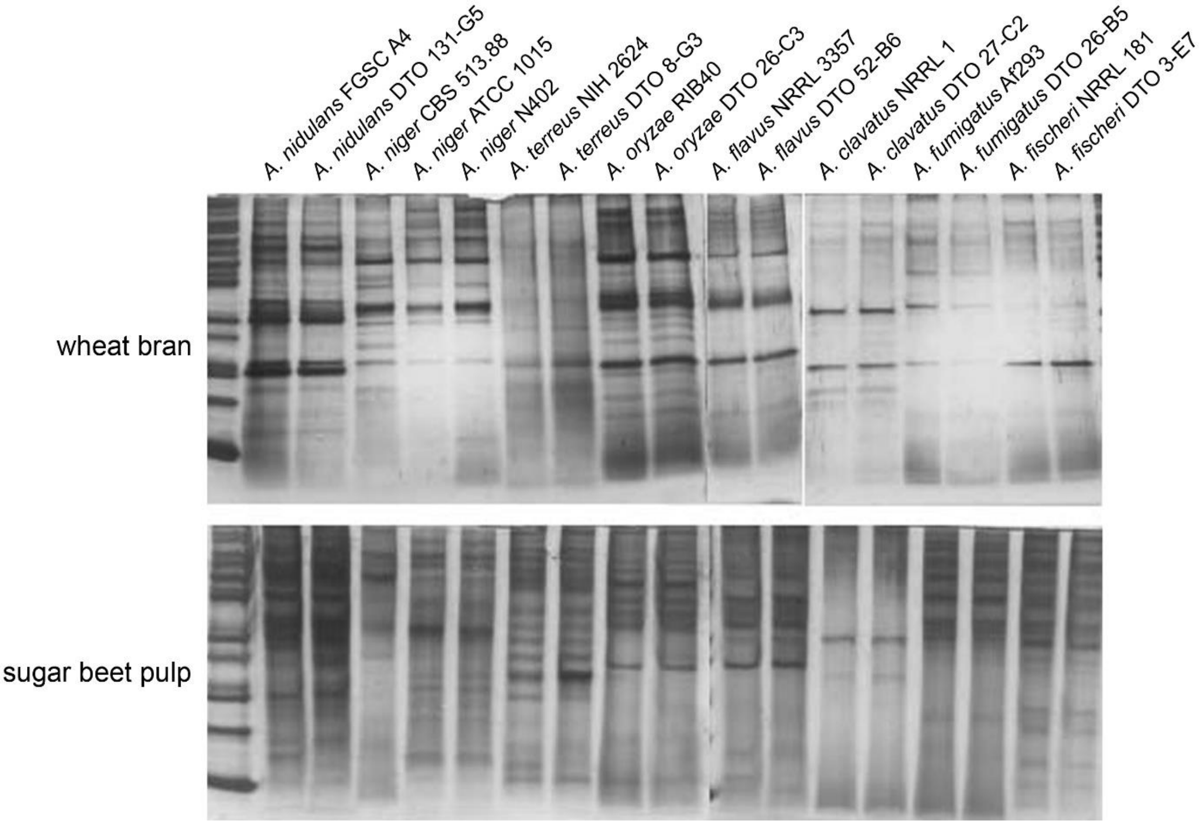

SDS-PAGE profiles of the strains used in this study.

Extracellular culture samples from the wheat bran and sugar beet cultures were separated by SDS-PAGE and the gels were stained using silver staining. Two or three isolates per species were analysed, which demonstrated high conservation of the extracellular protein profile within a species. Larger differences were visible between the species. The profiles of more closely related species (*A. oryzae – A. flavus* and *A. fumigatus – A. fischeri*) were more similar to each other than to the profiles of the other species.

Supplemental Table 1. Strains used in this study

Species	Strain number	Alternative strain number	Genome sequence reference
A. nidulans	FGSC A4	ATCC 38163	[2]
A. nidulans		DTO 131-G5	n/a
A. niger	CBS 513.88		[3]
A. niger	ATTC 1015	CBS 113.46, NRRL 328, FGSC A1144	[4]
A. niger	N402		n/a
A. terreus	NIH 2624	FGSC A1156	unpublished
A. terreus		DTO 8-G3	n/a
A. oryzae	RIB 40	ATCC 42149	[5]
A. oryzae		DTO 26-C3	n/a
A. flavus	NRRL 3357	CBS 128202, ATCC 200026	[6]
A. flavus		DTO 52-B6	n/a
A. clavatus	NRRL 1	CBS 513.65, ATCC 1007	[7]
A. clavatus		DTO 27-C2	n/a
A. fischeri	NRRL 181	CBS 544.65, ATCC 1020	[7]
A. fischeri		DTO 3-E7	n/a
A. fumigatus	Af293	FGSC A1435	[8]
A. fumigatus		DTO 26-B5	n/a

n/a = not available

References

1. de Vries RP, Burgers K, van de Vondervoort PJI, Frisvad JC, Samson RA, Visser J: **A new black *Aspergillus* species, *A. vadensis*, is a promising host for homologous and heterologous protein production.** *Appl Environ Microbiol* 2004, **70:**3954-3959.
2. Galagan JE, Calvo SE, Cuomo C, Ma LJ, Wortman JR, Batzoglou S, Lee SI, Basturkmen M, Spevak CC, Clutterbuck J, et al: **Sequencing of *Aspergillus nidulans* and comparative analysis with *A. fumigatus* and *A. oryzae*.** *Nature* 2005, **438:**1105-1115.
3. Pel HJ, de Winde JH, Archer DB, Dyer PS, Hofmann G, Schaap PJ, Turner G, de Vries RP, Albang R, Albermann K, et al: **Genome sequencing and analysis of the versatile cell factory *Aspergillus niger* CBS 513.88.** *Nat Biotechnol* 2007, **25:**221-231.
4. Andersen MR, Salazar MP, Schaap PJ, van de Vondervoort PJ, Culley D, Thykaer J, Frisvad JC, Nielsen KF, Albang R, Albermann K, et al: **Comparative genomics of citric-acid-producing *Aspergillus niger* ATCC 1015 versus enzyme-producing CBS 513.88.** *Genome Res* 2011, **21:**885-897.
5. Machida M, Asai K, Sano M, Tanaka T, Kumagai T, Terai G, Kusumoto K, Arima T, Akita O, Kashiwagi Y, et al: **Genome sequencing and analysis of *Aspergillus oryzae*.** *Nature* 2005, **438:**1157-1161.
6. Yu J, Payne GA, Nierman WC, Machida M, Bennett JW, Campbell BC, Robens JF, Bhatnagar D, Dean RA, Cleveland TE: ***Aspergillus flavus* genomics as a tool for studying the mechanism of aflatoxin formation.** *Food Addit Contam Part A Chem Anal Control Expo Risk Assess* 2008, **25:**1152-1157.
7. Fedorova ND, Khaldi N, Joardar VS, Maiti R, Amedeo P, Anderson MJ, Crabtree J, Silva JC, Badger JH, Albarraq A, et al: **Genomic islands in the pathogenic filamentous fungus *Aspergillus fumigatus*.** *PLoS Genet* 2008, **4:**e1000046.
8. Nierman WC, Pain A, Anderson MJ, Wortman JR, Kim HS, Arroyo J, Berriman M, Abe K, Archer DB, Bermejo C, et al: **Genomic sequence of the pathogenic and allergenic filamentous fungus *Aspergillus fumigatus*.** *Nature* 2005, **438:**1151-1156.
